# Experiment and simulation of single inhibitor SH110 for void-free TSV copper filling

**DOI:** 10.1038/s41598-021-91318-9

**Published:** 2021-06-08

**Authors:** Fuliang Wang, Yuping Le

**Affiliations:** 1 School of Mechanical and Electrical Engineering, Central South University, Changsha, 410083 HN Province People’s Republic of China; 2 State Key Laboratory of High Performance Complex Manufacturing, Changsha, 410083 People’s Republic of China; 3 School of Mechanical and Electrical Engineering, Guilin University of Electrical Technology, Guilin, People’s Republic of China

**Keywords:** Chemistry, Materials science

## Abstract

Three-dimensional integration with through-silicon vias (TSVs) is a promising microelectronic interconnection technology. Three-component additives are commonly used for void-free TSV filling. However, optimising the additive concentrations is an expensive process. To avoid this, a single-component additive was developed: 3-(2-(4,5-dihydrothiazol-2-yl) disulfanyl) propane-1-sulfonic acid/sulfonate (SH110). Sodium 3,3′-dithiodipropane sulfonate (SPS) and SH110 were used as additives for TSV electroplating copper filling. SH110 resulted in void-free filling, whereas large keyhole voids were found for SPS. To understand how the additives affect the filling mechanism, linear sweep voltammetry of the plating solutions was carried out. The interactions between the Cu surface and additives were simulated by molecular dynamics (MD) analysis using Materials Studio software, and quantum chemistry calculations were conducted using GAUSSIAN 09W. SH110 adsorbs to the Cu surface by both 4,5-dihydrothiazole (DHT) and 3-mercaptopropane sulfonate (MPS) moieties, while SPS is adsorbed only by MPS moieties. MD simulations indicated that the adsorption of the coplanar MPS moiety is the main factor governing acceleration. Quantum chemistry calculations showed that DHT provides an inhibitory effect for TSV filling, while MPS acts as an accelerator for SH110. SH110 is an excellent additive exhibiting both the acceleration and the suppression necessary for achieving void-free TSV filling.

## Introduction

Three-dimensional (3D) integration with through-silicon vias (TSVs) is a promising technology for use in electronic systems, as TSVs can provide extremely short vertical interconnections that can improve performance, increase operating speed, and reduce the volume of devices when compared with conventional integration technologies^1–3^. TSV copper filling is one of the key techniques used for TSV fabrication, as it costs ~ 40% less than conventional integration technologies. However, voids often occur upon filling, which must be overcome for reliable TSV fabrication^4,5^.

To accomplish void-free TSV filling, an accelerator, suppressor, and leveller are commonly added to the plating solution. At present, the most commonly used accelerator is sodium 3,3′-dithiodipropane sulfonate (SPS)^6^, where the sulfur S–S bonds and sulfonic acid or sulfonate groups (SO_3_H or SO_3_^–^) are thought to be the key structures responsible for acceleration effects. The typical leveller employed is an organic monomer containing positively charged nitrogen, such as pyridinium, imidazolium, or ammonium^7,8^. Polyethylene glycol (PEG), polypropylene glycol (PPG), and co-polymers thereof are commonly used as suppressor additives^9,10^. These additives accelerate the via bottom deposition rate and suppress the via mouth deposition rate, to obtain bottom-up filling. However, it takes numerous experiments to optimise the concentrations of each component in this complex additive system. Therefore, a single inhibitor that accomplishes void-free TSV filling is needed in order to reduce the time and cost of the optimisation process. Thus far, only one report, by Tang and co-workers, has demonstrated a single-component additive (Janus Green B) that could provide void-free filled-in micro-vias^11^.

In this study, a single inhibitor, 3-(2-(4,5-dihydrothiazol-2-yl)disulfanyl)propane-1-sulfonic acid (SH110), was found to possess the ability to fill the TSV without introducing voids. Linear sweep voltammetry (LSV) was performed to determine the electrochemical properties of additives in the deposition process for TSV filling. To understand how SH110 affects the TSV filling mechanism, molecular dynamics (MD) simulations and quantum chemical calculations were used to analyse the configuration and electronic structure of SH110 and its interactions with the copper surface, in comparison with a common accelerator, SPS.

## Results and discussion

### Electroplating

**Figure 1:**
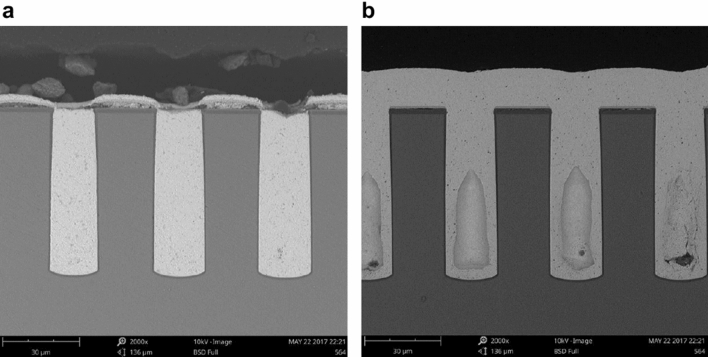
Vertical cross-sectional SEM image of TSV filling results with accelerators. (**a**) SH110 and (**b**) SPS (TESCAN VEGA 3 https://www.tescan.com/).

SH110 and SPS were separately used as additives for TSV electroplating. Vertical cross-sectional scanning electron microscopy (SEM) images of the filled TSVs are shown in Fig. 1a,b. The applied electroplating current density was 1 mA/cm^2^ with plating times of 12 h. Using SH110 as the additive, the TSVs were fully filled without any voids, as shown in Fig. 1a. The thin film on top of the silicon indicates that these vias were filled in a bottom-up manner. However, for the sample with SPS as the additive, large keyhole voids were found, as shown in Fig. 1b. Thus, SH110 provides excellent filling behaviour.

**Figure 2:**
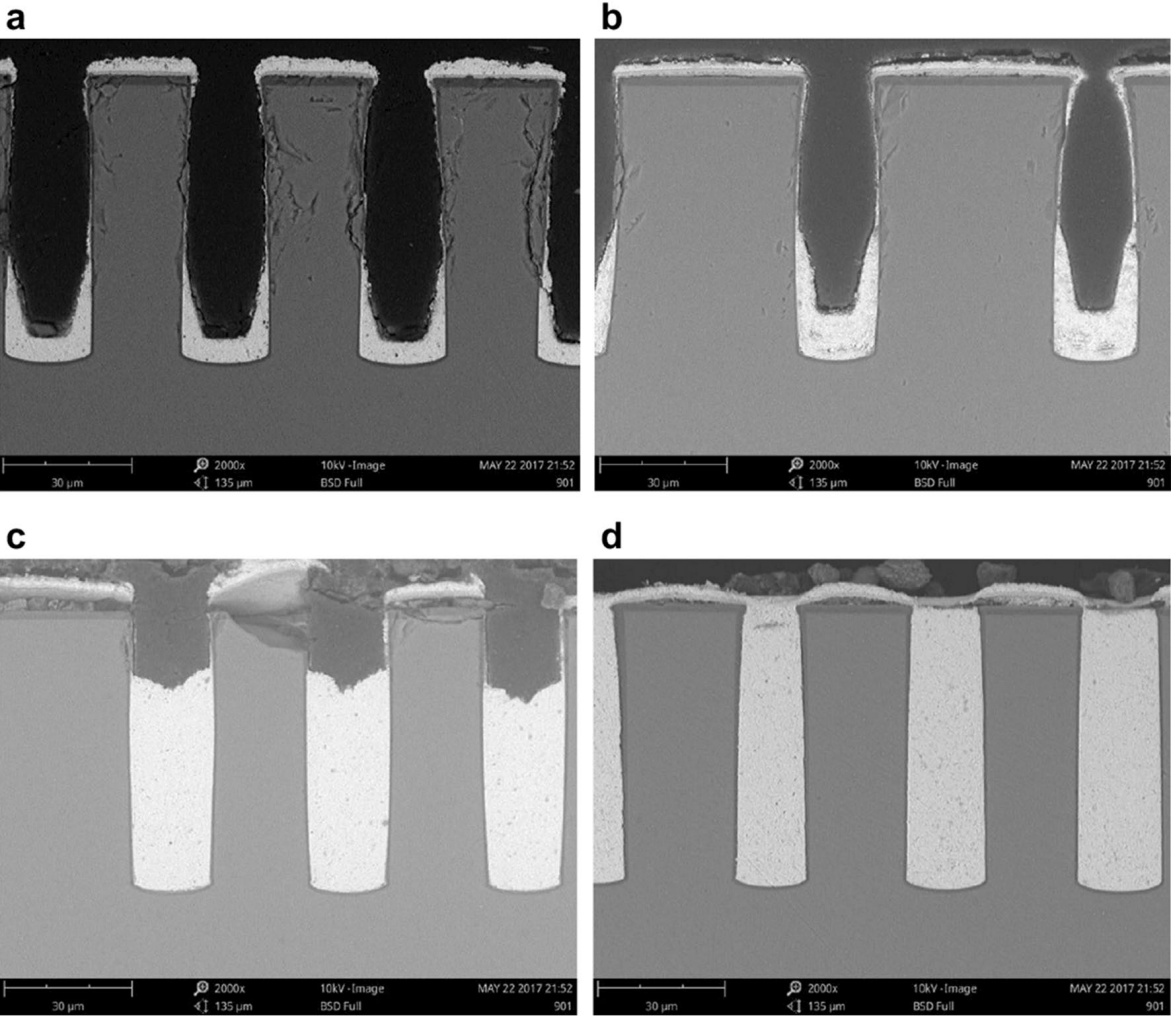
Vertical cross-sectional SEM images of TSVs filled using SH110 additive at different times. (**a**) 2 h, (**b**) 3 h, (**c**) 8 h, and (**d**) 12 h (TESCAN VEGA 3 https://www.tescan.com/).

To better understand how the SH110 additive facilitated void-free TSV filling, TSVs filled at different plating times were obtained, as shown in Fig. 2. (Note that, as we designed five different pitches for the vias in the same die, the pitches of the vias in Fig. 2 vary.) Initially (2 h, Fig. 2a), the TSVs were only plated at the bottom, following the U-shaped model; copper deposition was almost totally suppressed in the top half of the vias. Over time (3 h, Fig. 2b), the thickness of the copper at the bottom half of the vias increased, whereas the top half of the via remained suppressed. Then, after the bottom half of the vias were completely filled (8 h, Fig. 2c), copper began to be deposited in the top half in a bottom-up manner. The entire vias were fully filled after 12 h (Fig. 2d).

Therefore, with the single inhibitor SH110, the TSVs were filled according to different models at different stages in the filling process. The first stage is the filling of the bottom half of the vias, which proceeds by the U-shape model; the top half of the vias are filled in the second stage, following the bottom-up model. It should be noted that the thickness of the deposited copper layer at the surface remained unchanged with time, indicative of total suppression of the surface.

Figures 1 and 2 is generate by Scanning electron microscope (SEM) (TESCAN VEGA 3 https://www.tescan.com/). SEM is used to observe the cross section of TSV copper plating filling. The TSV hole was magnified by 2000–3000 times. Electron microscope was used to take photos directly.

### Electrochemical procedures

**Figure 3:**
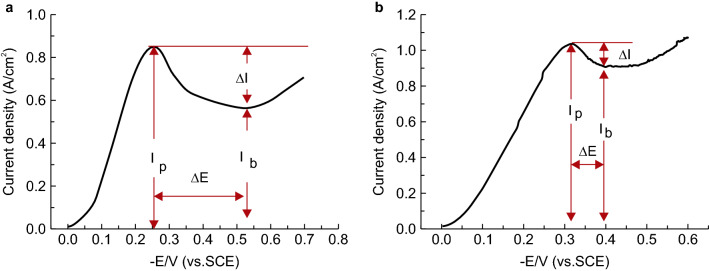
LSV measured in the plating bath. (**a**) Plating solution with SH110 and (**b**) plating solution with SPS.

To understand how the additives affect the filling mechanism for TSVs, LSVs was performed using plating solutions with either SH110 or SPS additives, as shown in Fig. 3 and Table 1. According to the additive theory, a large peak current density (I_p_) means acceleration plays a leading role, whereas a large valley (I_b_) suggests that suppression is predominant. Thus, a larger ΔI value (I_p_ − I_b_) indicates better bottom-up filling ability in TSVs, and a larger ΔE (the potential gap between I_p_ and I_b_) indicates that there is a wider potential region accessible for bottom-up filling^12,13^.

**Table 1:** Liner sweep voltammetry (LSV) results of SPS and SH110 electroplating solutions.

	SH110	SPS
I_p_ (A/cm^2^)	0.859	1.030
I_b_ (A/cm^2^)	0.567	0.910
E_p_	− 0.250	− 0.314
E_b_	− 0.521	− 0.397
ΔI (A/cm^2^)	0.292	0.120
ΔE (V)	0.271	0.073

For the primary solution containing SH110, the peak current density (I_p_ = 0.859 A/cm^2^) appears at approximately − 0.250 V, with a valley (I_b_ = 0.567 A/cm^2^) at approximately − 0.521 V. For the plating solution with SPS, the peak current density (I_p_ = 1.03 A/cm^2^) appears at approximately − 0.314 V, with a valley (I_b_ = 0.91 A/cm^2^) at approximately − 0.397 V. Moreover, the ΔI and ΔE values of the solution with SH110 are 0.292 A/cm^2^ and 0.271 V, respectively, which are both greater than those with SPS (0.12 A/cm^2^ and 0.073 V, respectively).

Therefore, SH110 provides better bottom-up filling ability than SPS, as it has better suppression effects at high-potential locations, such as the via mouth. The larger ΔE of SH110 also means it has a wider potential operation window than SPS. Employing SH110 as a solitary additive results in a fully filled TSV, while the SPS additive results in keyhole voids (Fig. 1).

### Molecular dynamics simulation

Since the adsorption characteristics of additives are intrinsically linked to their role in TSV filling, MD simulations were performed to study the adsorption behaviour of SPS and SH110 on Cu (001), Cu (101) and Cu (111) surface. Because SPS and SH110 are involved in the filling process of electroplated copper, the surfaces most likely to be contacted are the surface of Cu (111), the surface of Cu (111) and the surface of copper during the filling process of electroplated copper, the main consideration in the filling process of TSV is (111) .

**Figure 4:**
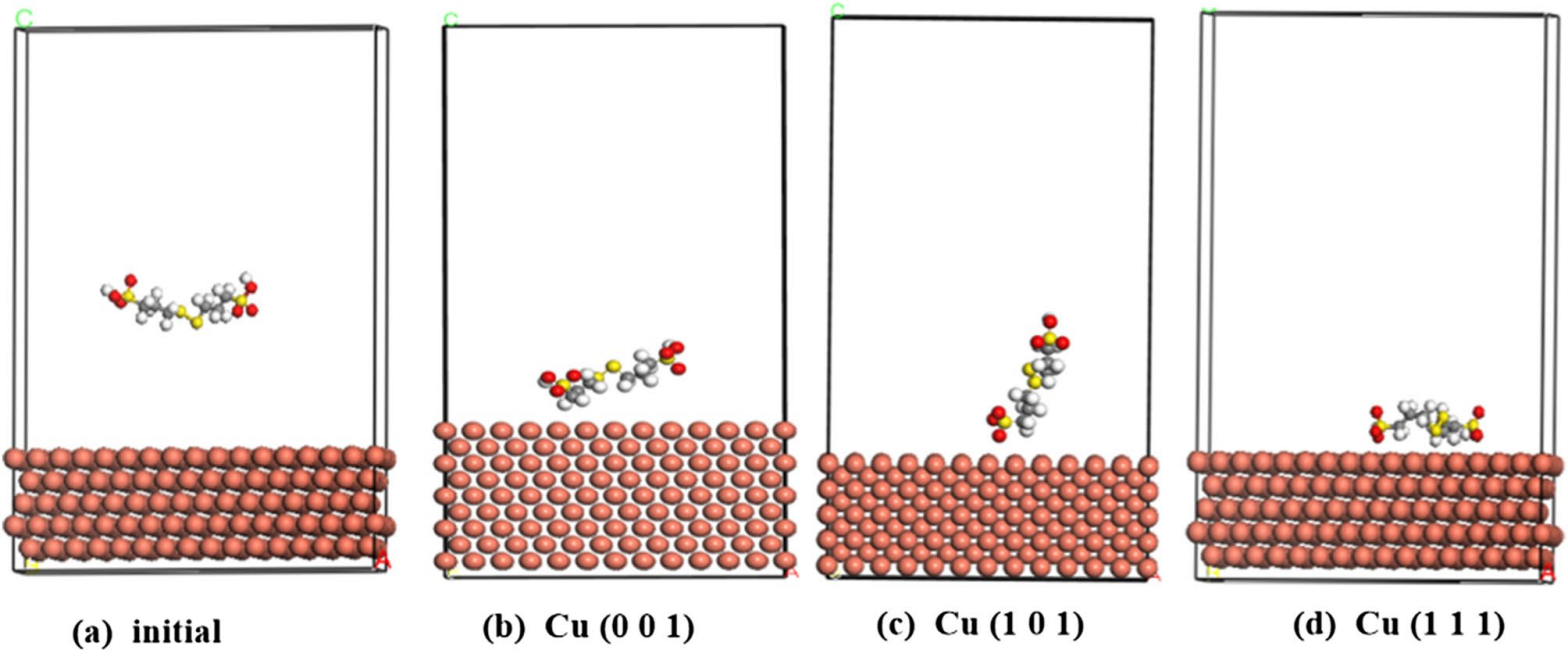
Configuration of SPS adsorbed on Cu (001), Cu (101) and Cu (111) surface. (**a**) Side view of initial stage on Cu (111), (**b**) side view of equilibrium stage on Cu (001) surface (**c**) side view of equilibrium stage on Cu (101) surface. (**d**) side view of equilibrium stage on Cu (101) surface (https://www.3ds.com/products-services/biovia/ material studio 6.0).

Figure 4a–d show the initial and equilibrium stages for adsorption of SPS. It can be seen that In the initial state onCu (111) (Fig. 4a), the MPS groups are oriented away from the copper surface, thus giving the SPS molecule a V-shape. SPS is adsorbed coplanar to the copper surface when in equilibrium on Cu (001) (Fig. 4b). SPS is vertically adsorbed on the surface of the copper crystal by sulfonic group(SO_3_H) when in equilibrium on Cu (101) (Fig. 4c). SPS is adsorbed coplanar to the copper surface by the two MPS groups when in equilibrium on Cu (111) (Fig. 4d). Since SPS is a commonly employed accelerator, our results suggest that the coplanar adsorption of MPS groups to the copper surface is the main factor contributing to acceleration^10,14–16^.

**Figure 5:**
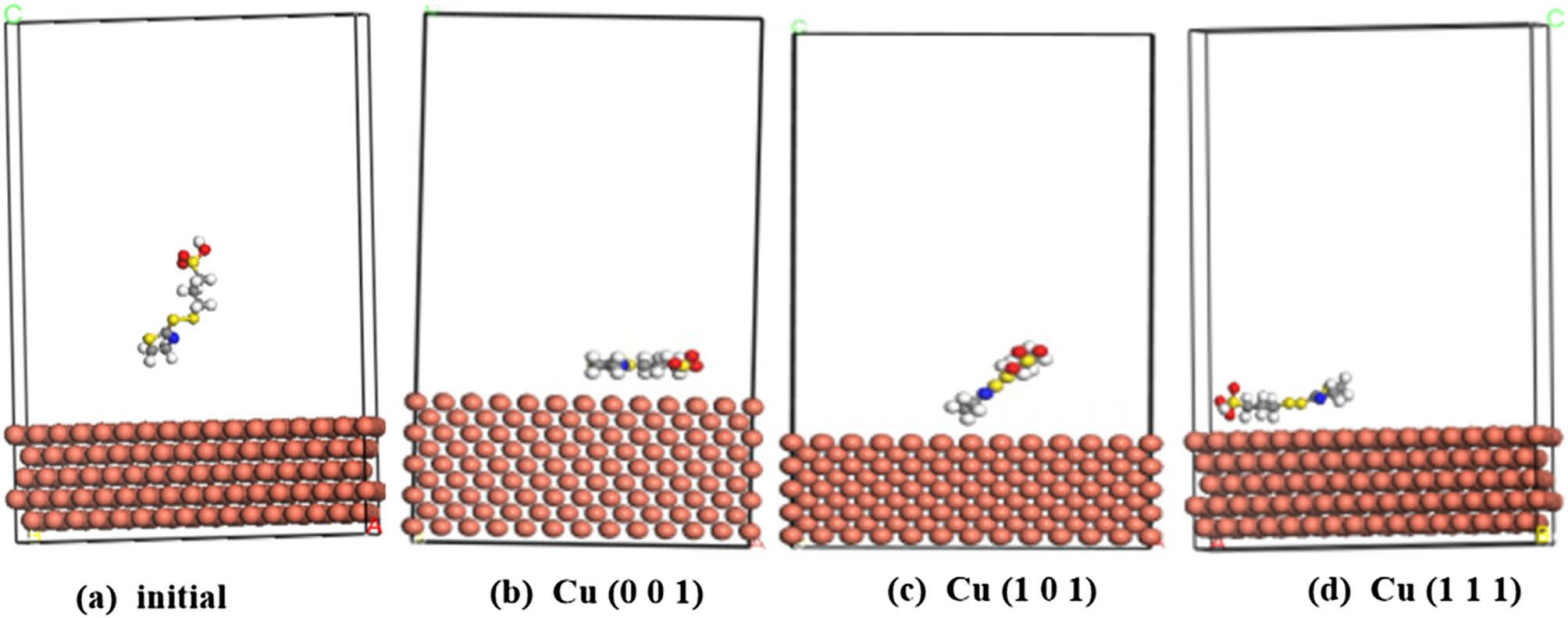
Configuration of SH110 adsorbed on Cu (001), Cu (101) and Cu (111) surface. (**a**) Side view of initial stage on Cu (111), (**b**) side view of equilibrium stage on Cu (001) surface (**c**) side view of equilibrium stage on Cu (101) surface. (**d**) side view of equilibrium stage on Cu (101) surface (https://www.3ds.com/products-services/biovia/ material studio 6.0).

Figure 5a–d show the initial and equilibrium stages for adsorption of SH110. Initially (Fig. 5a), SH110 is located far from the copper surface, suggesting only very slight adsorption. It is vertically oriented and positioned in a sideways V-shape. In equilibriumon, Similar to SPS, SH110 is adsorbed coplanar to the Cu(001) surface (Fig. 5b). SH110 is Vertically adsorbed on the Cu(101)surface of the copper crystal by the DHT moiety SH110 is adsorbed coplanar to the copper surface when in equilibrium, but at three points on the molecule: the DHT moiety, the S–S bond, and the –SO_3_^−^ group. The DHT moiety is oriented closest to the surface of the copper, making this group the dominant contributor to this initial (but slight) adsorption.

The equilibrium adsorption of S–S and –SO_3_^−^ groups on the copper surface contribute to the acceleration associated with SH110 as an additive. Meanwhile, the DHT group adsorbed to the copper surface contributes to SH110 having an inhibitory effect. Thus, we can conclude that the DHT, S–S, and –SO_3_^−^ groups are crucial to SH110 adsorption behaviour, which results in fully filled TSVs.

### Quantum chemistry calculation

#### Equilibrium geometry structure

**Figure 6:**
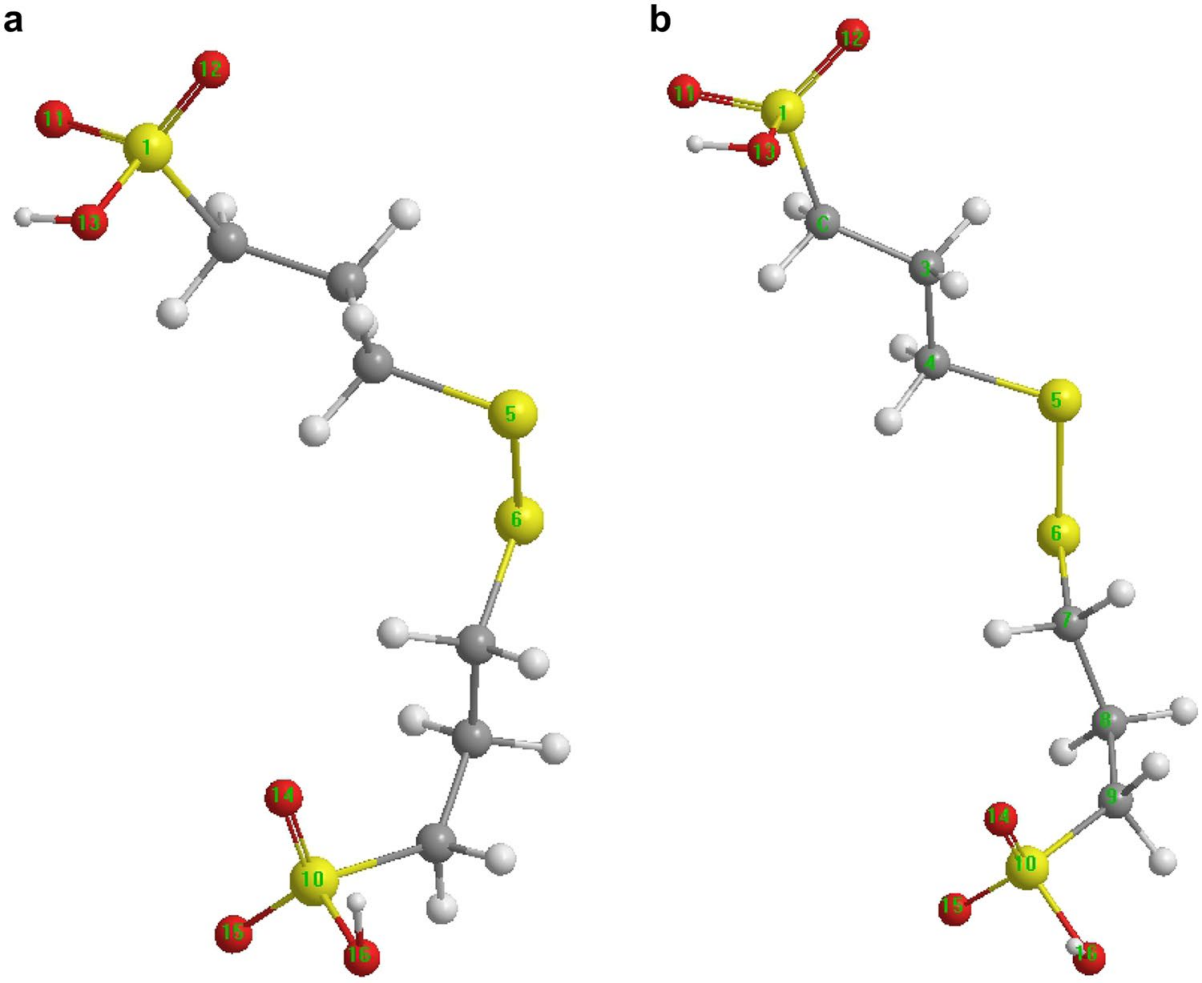
Molecular configuration of SPS from MD simulations. (**a**) Initial and (**b**) equilibrium structures (https://www.3ds.com/products-services/biovia/ material studio 6.0).

**Figure 7:**
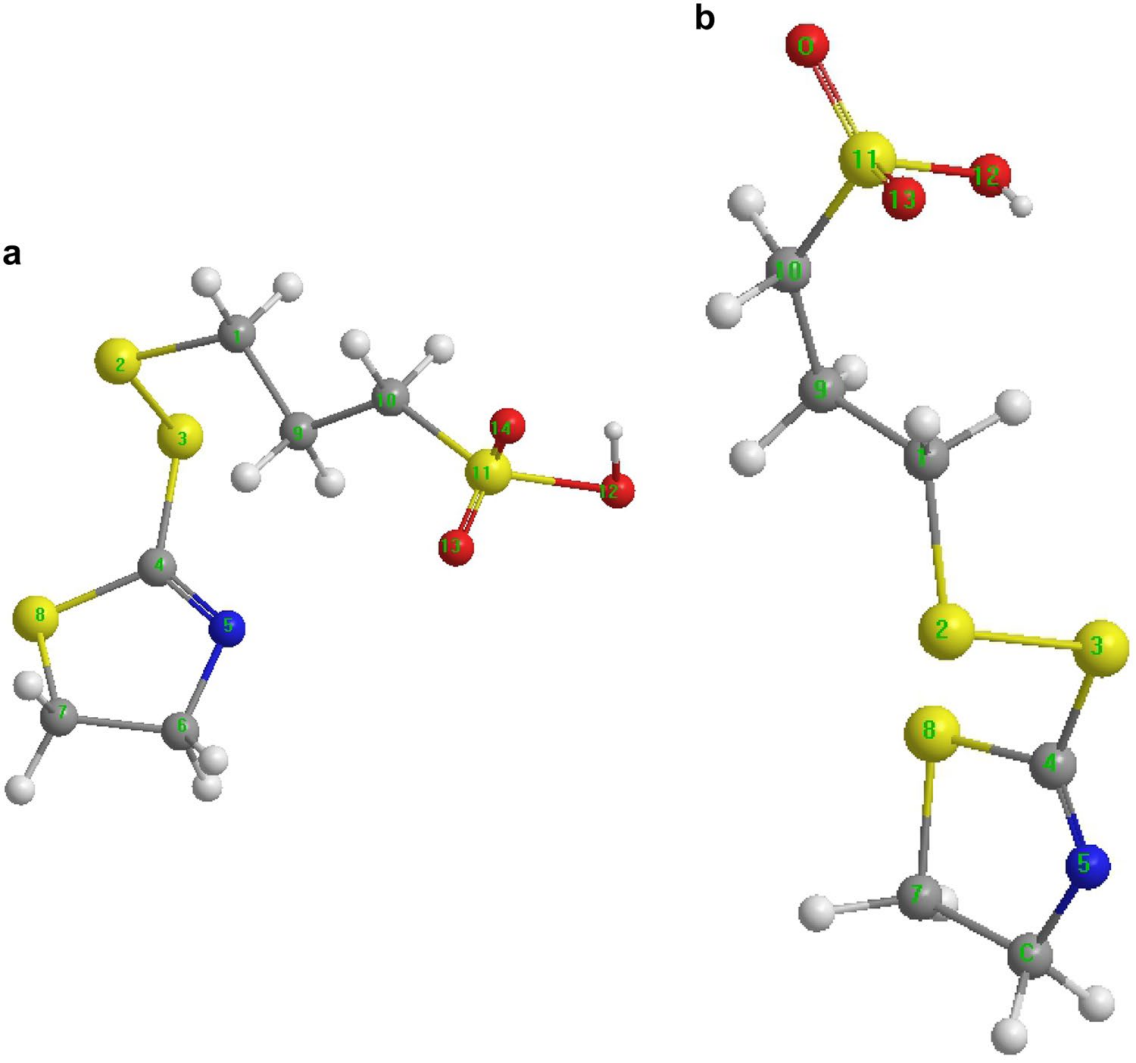
Molecular configuration of SH110 from MD simulations. (**a**) Initial and (**b**) equilibrium structures (https://www.3ds.com/products-services/biovia/ material studio 6.0).

The initial and equilibrium structures of SPS were obtained from MD simulations. To further study how the additives were adsorbed on the copper surface, the initial and adsorbed equilibrium structures of SPS and SH110 were obtained by the DFT B3LYP method, as shown in Figs. 6 and 7.

For SPS in Fig. 6, atoms 1, 5, 6, and 10 are sulfur, while 11–16 are oxygen, and 2, 3, 4, 7, 8, and 9 are carbon. For SH110 in Fig. 7, atoms 2, 3, 8, and 11 are sulfur, 12–14 are oxygen, 5 is nitrogen, and 1, 4, 6, 7, 9, and 10 are carbon.

The optimised bond lengths of SPS and SH110 in initial and equilibrium conditions on the Cu surface were also obtained. For SPS (Table 2), most bonds became longer in equilibrium compared to the initial state, except for C9–S10. It should be noted that the S–O bonds changed the most (~ 5%); this suggests that the SO_3_^−^ group strongly interacts with the Cu surface. The changes in bond lengths that occur for SH110 from initial to equilibrium adsorption are also given in Table 2. For instance, C4–N5 shortens by ~ 13% upon adsorption, suggesting a strong interaction between the DHT moiety and the copper surface. The C10–S11 bond length also shortens by ~ 6% upon adsorption, suggesting that the interaction between the MPS group and copper is also important.

**Table 2:** Selected bond lengths for SH110 and SPS.

SPS	Bond length			SH110	Bond length		
	Initial (nm)	Equilibrium (nm)	Change (%) (Equilibrium/Initial)		Initial (nm)	Equilibrium (nm)	Change (%) (Equilibrium/Initial)
S1–C2	0.2606	0.2645	1.4965464	C1–S2	0.2607	0.2659	1.9946298
S1=O11	0.1974	0.2035	3.0901722	C1–C9	0.2269	0.2209	− 2.6443367
S1=O12	0.1971	0.2027	2.8411974	S2–S3	0.3039	0.3052	0.4277723
S1–O13	0.2288	0.2307	0.8304196	S3–C4	0.2505	0.2418	− 3.4730539
C2–C3	0.2206	0.223	1.087942	C4–N5	0.1918	0.1677	− 12.5651721
C3–C4	0.2222	0.2287	2.9252925	C4–S8	0.2487	0.2469	− 0.7237636
C4–S5	0.2604	0.2623	0.7296467	N5–C6	0.2133	0.2075	− 2.7191749
S5–S6	0.2934	0.3012	2.6584867	C6–C7	0.2275	0.2246	− 1.2747253
S6–C7	0.2609	0.2676	2.5680337	C7–S8	0.2565	0.2532	− 1.2865497
C8–C9	0.2226	0.2269	1.9317161	C9–C10	0.2186	0.2319	6.084172
C9–S10	0.2693	0.2598	− 3.5276643	C10–S11	0.2617	0.2528	− 3.4008407
S10–O14	0.2036	0.2143	5.2554028	S11–O12	0.2278	0.2243	− 1.5364355
S10–O15	0.1972	0.2075	5.2231237	S11–O13	0.2015	0.1911	− 5.1612903
S10–O16	0.2168	0.2226	2.6752768	S11–O14	0.2011	0.1961	− 2.4863252

#### Active sites

To reveal how the groups on each additive interact with the copper surface, an active site was established according to two factors: (1) natural atomic charge and (2) distribution of the frontier molecular orbitals^17^. This was accomplished by using quantum chemical calculations of the orbital information and the electronic properties.

1. Natural atomic charge

Table 3 shows the natural atomic charges of SPS and SH110 initially and at equilibrium. For SPS, O11, O12, O14, and O15 carry larger negative charges in the initial state than at equilibrium, and all other atoms exhibit very little change from the initial to equilibrium states. This suggests that O11, O12, O14, and O15 have negative charge centres that could offer electrons to the Cu atoms to form coordinate bonds that are initially strong, but weaken at equilibrium. S1 and S10 carry large positive charges both in the initial state and at equilibrium, indicating that they possess positive charge centres that can accept electrons from the 3*d* orbital of the Cu atoms to form a feedback bond in both states, although this bond would also be weaker in equilibrium than the initial state. This suggests that SO_3_^−^ is the driving force of the electrostatic interactions and chemical properties of SPS interacting with the Cu surface. This is one reason SO_3_^−^ is often present in compounds used as accelerators.

**Table 3:** Natural atomic charge of SPS and SH110 initially and at equilibrium.

SPS atom	Charge (C)		ΔC	SH110 atom	Charge (C)		ΔC
	Initial	Equilibrium			Initial	Equilibrium	
S1	0.5466	0.4703	0.0763	C1	0.0779	0.0488	0.0291
C2	0.0309	0.0426	− 0.0117	S2	− 0.0577	− 0.0594	0.0017
C3	0.0956	0.095	0.0006	S3	− 0.0202	− 0.0071	− 0.0131
C4	0.073	0.0835	− 0.0105	C4	− 0.0093	0.0174	− 0.0267
S5	− 0.0758	− 0.0815	0.0057	N5	− 0.0913	− 0.1448	0.0535
S6	− 0.0889	− 0.0921	0.0032	C6	0.0762	0.0992	− 0.023
C7	0.0934	0.0974	− 0.004	C7	0.0737	0.0543	0.0194
C8	0.0956	0.1064	− 0.0108	S8	− 0.0559	− 0.0585	0.0026
C9	0.0417	0.0467	− 0.005	C9	0.1224	0.0958	0.0266
S10	0.5408	0.4154	0.1254	C10	0.0283	0.0654	− 0.0371
O11	− 0.3246	− 0.2967	− 0.0279	S11	0.5004	0.6034	− 0.103
O12	− 0.2907	− 0.2649	− 0.0258	O12	− 0.0614	− 0.0749	0.0135
O13	− 0.0661	− 0.0554	− 0.0107	O13	− 0.2733	− 0.3456	0.0723
O14	− 0.3125	− 0.2587	− 0.0538	O14	− 0.31	− 0.2938	− 0.0162
O15	− 0.2864	− 0.2534	− 0.033				
O16	− 0.0728	− 0.0545	− 0.0183				

For SH110, N5, O13, and O14 carried larger negative charges in the initial state than at equilibrium. This indicates that N5, O13, and O14 are negative charge centres that can offer electrons to the Cu atoms to form coordinate bonds. Moreover, the bonds with N5 and O13 are stronger at equilibrium than in the initial state. S11 carries a positive charge, which indicates a positive charge centre that can accept electrons from the 3*d* orbital of the Cu atoms to form coordinate covalent bonds. These S11 bonds are also stronger at equilibrium than in the initial state, further strengthening the interaction of SH110 with the Cu surface.

2. Distribution of the frontier molecular orbitals

**Figure 8:**
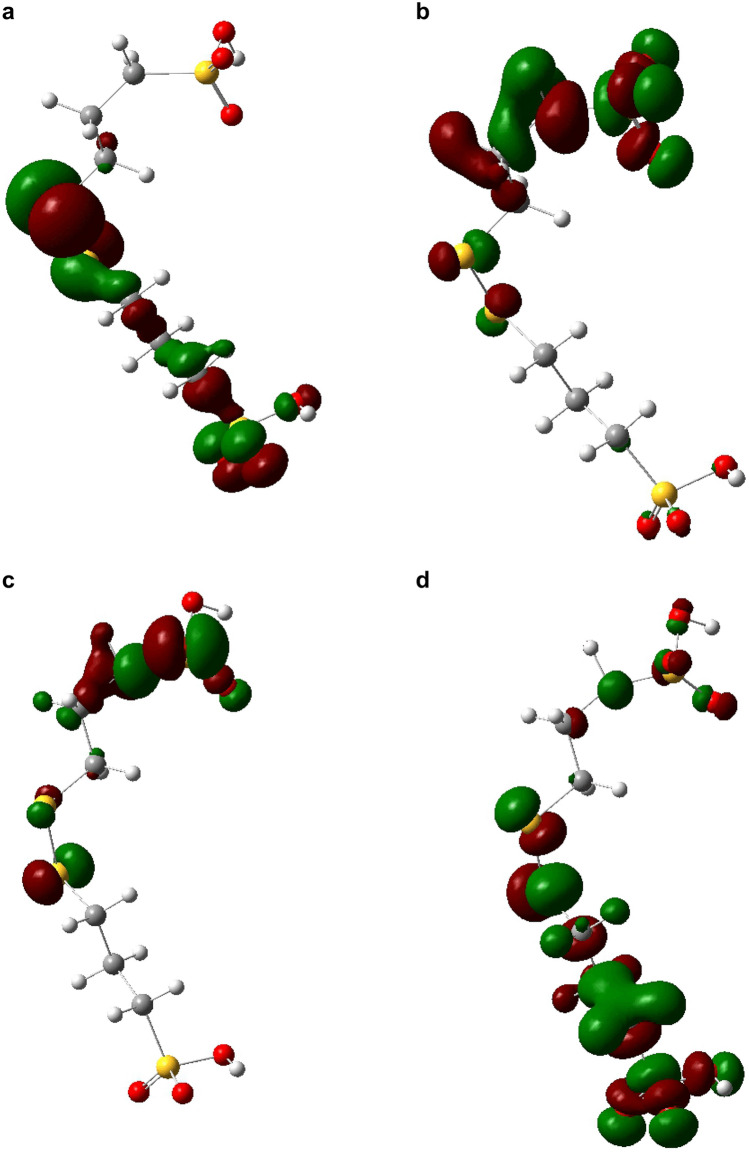
Geometry of SPS. (**a**) Initial HOMO, (**b**) initial LUMO, (**c**) equilibrium HOMO, and (**d**) equilibrium LUMO (https://www.3ds.com/products-services/biovia/ material studio 6.0).

Figure 8 shows the highest occupied and lowest unoccupied molecular orbitals (HOMO and LUMO, respectively) of SPS in both the initial and equilibrium states. We can see that SPS essentially comprises two MPS molecules. The main components of the molecular HOMO and LUMO orbitals are listed in Table 4.

**Table 4:** Components of SPS molecular orbitals.

SPS	HOMO (%)		LUMO (%)	
Stage	Initial	Equilibrium	Initial	Equilibrium
S1	0.74	0	0.21	11.45
C2	5.74	0.03	0.39	2.62
C3	1.23	0.01	0.20	9.59
C4	2.14	0.03	0.14	8.21
S5	10.76	5.51	1.57	12.43
S6	50.50	1.44	2.53	7.06
C7	0.30	0.38	0	0.37
C8	0.05	1.43	9.40	1.30
C9	0.05	10.62	29.52	2.01
S10	0.01	0.87	13.26	0.02
O11	11.51	0.07	0	11.81
O12	13.38	0.08	0.19	11.36
O13	1.48	0	0.14	7.81
O14	0.14	2.65	10.74	1.21
O15	0.14	76.64	13.55	0.89
O16	0.01	0.17	7.79	0.61

In the initial stage of adsorption, S5, S6, O11, O12, and O13 contribute 86.2% to the HOMO, with S6 contributing the most (50.50%). This indicates that the atoms in S–S and SO_3_^−^ play a major role in governing the chemical reactions and can interact strongly with the copper surface. The LUMO is mostly comprised of contributions from C8, C9, S10, O14, O15, and O16, at 9.40%, 29.52%, 13.26%, 10.74%, 13.55%, and 7.79%, respectively. This indicates that SPS can accept electrons from the 3*d* orbitals of Cu atoms, thus further strengthening the interaction between SPS and the copper surface.

After adsorption, the frontier molecular orbitals are redistributed. The HOMO is mainly comprised of O15, C9, and S5. This indicates that O15, C9, and S5 can offer electrons; O15 has the maximum electrophilic electron density for charge transfer (76.64%). Thus, SO_3_^−^ strongly adsorbs to the copper surface. S1, C3, C4, S5, S6, O11, O12, and O13 contribute 90% of the LUMO, indicating that electrons can be accepted from the 3*d* orbitals of Cu atoms, further strengthening the interaction of SPS with the copper surface. SPS mainly interacts with the copper surface through the S and SO_3_^−^ groups. This may be the reason that SPS is an excellent additive for acceleration.

**Figure 9:**
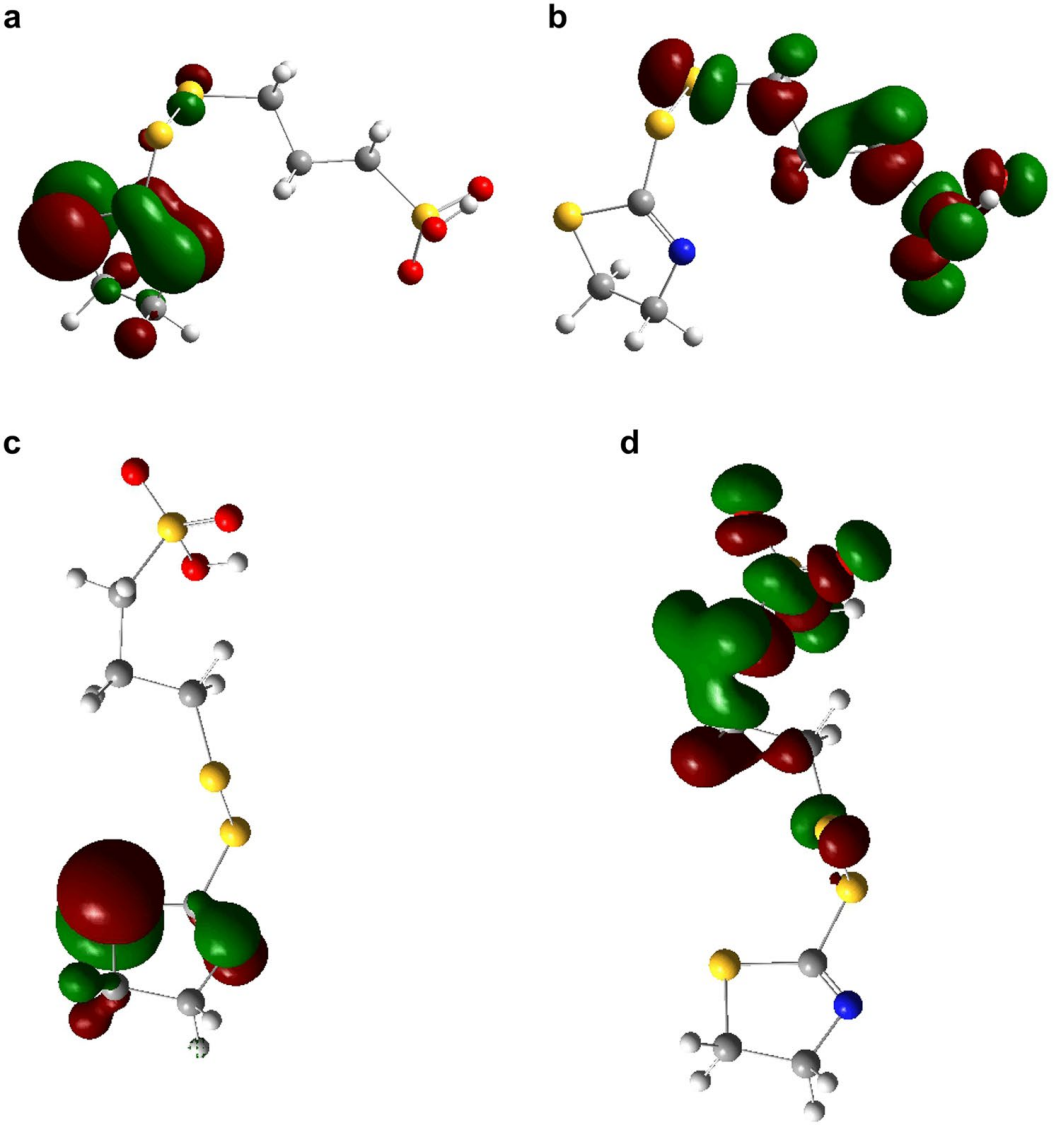
Geometry of SH110. (**a**) Initial HOMO, (**b**) initial LUMO, (**c**) equilibrium HOMO, and (**d**) equilibrium LUMO (https://www.3ds.com/products-services/biovia/ material studio 6.0).

Figure 9 shows the HOMO and LUMO orbitals of the initial and equilibrium states of SH110. The main components of the HOMO and LUMO orbitals are listed in Table 5. In the initial stage of adsorption, N5 and S8 contribute over 87.74% to the HOMO. Thus, N5 and S8 can offer electrons. In addition, the DHT moiety plays a major role in governing chemical reactions and can interact strongly with the copper surface. The LUMO is mainly comprised of C1, S2, C9, C10, S11, O12, O13, and O14. This indicates that all these atoms have some ability to accept electrons from the 3*d* orbitals of Cu atoms, thus further strengthening the interaction of SH110 with the copper surface. DHT is dominant in the initial stage of adsorption (as shown in Fig. 5), while SO_3_^−^ has no ability to donate electrons, which differs considerably from SPS. After adsorption to the copper surface, the frontier molecular orbitals are redistributed. N5 and S8 from DHT contribute over 95.49% to the HOMO in equilibrium, while the atoms associated with the MPS moiety (C9, C10, S11, O12, O13, and O14) do not contribute at all to the HOMO. We can therefore conclude that the DHT moiety of SH110 donates electrons to Cu atoms and becomes strongly adsorbed to the copper surface. At equilibrium, N5 and S8 of the DHT moiety can interact more strongly with the copper surface, enabling SH110 to cover the copper surface and prevent further deposition. This may be the reason that SH110 is an excellent additive that provides an inhibitory effect. The MPS moiety (C1, S2, S3, C9, C10, S11, O12, O13, and O14) contributes to the LUMO, indicating that this portion of SH110 can accept electrons from the 3*d* orbitals of Cu atoms, further strengthening the interaction of SH110 with the copper surface. This is likely the reason that SH110 behaves as an excellent additive for acceleration. Therefore, SH110 is unique in that the DHT moiety provides an inhibitory effect for TSV filling, while the MPS moiety acts as an accelerator. SH110 exhibits an optimal balance between acceleration and suppression that allows for void-free TSV filling.

**Table 5:** Components of SH110 molecular orbitals.

SH110	HOMO (%)		LUMO (%)	
Stage	Initial	Equilibrium	Initial	Equilibrium
C1	0.01	0	8.31	1.41
S2	1.83	0.17	8.42	2.92
S3	0.71	0.48	0.33	0.51
C4	6.03	0.72	0.01	0.02
N5	28.06	9.55	0.01	0.04
C6	0.52	0.11	0	0
C7	0.51	0.43	0	0
S8	59.68	85.94	0	0
C9	0.014	0	7.89	11.09
C10	0	0	23.15	28.73
S11	0	0	12.18	13.55
O12	0	0	8.17	7.33
O13	0	0	11.31	9.09
O14	0	0	10.96	13.71

## Materials and methods

All chemical additives of SH110 and SPS were purchased from Jiangsumengde. SH110 and SPS are unstable in their acid forms, whereas the sodium salts are stable. Therefore, in the current study, we utilised the sodium salts of SPS and SH110 in acidic solution to obtain the corresponding acid in situ; the sodium salts were used for MD simulations as well.

**Figure 10:**
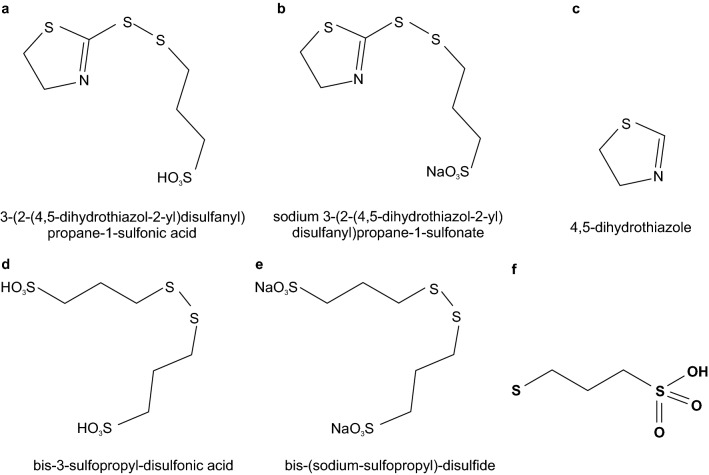
Chemical formulas of SH110 and SPS used in this study. (**a**) SH110; acidic form, (**b**) SH110; sodium salt, (**c**) 4,5-dihydrothiazole (DHT), (**d**) SPS; acidic form, (**e**) SPS; sodium salt, and (**f**) 3-mercaptopropane sulfonate (MPS).

The acidic and sodium salt forms of SH110 contain S–S bonds, SO_3_^−^ groups (with H^+^ or Na^+^, respectively), and DHT within their frameworks (Fig. 10a–c). Accelerators commonly contain S–S and SO_3_^−^^18^, and both are present in the acidic and sodium salt forms of SPS (Fig. 10d,e), which is a valuable accelerator^10,14–16^. Notably, DHT is commonly added during TSV filling as a leveller^7,8^, so the SH110 additive is expected to provide both accelerator and leveller properties. The MPS molecule in Fig. 10f corresponds to one half of a SPS molecule.

### Electroplating

The primary solution prepared for electroplating consisted of 0.78 mol/L CuSO_4_·5H_2_O, 0.2 mol/L H_2_SO_4_, and 0.2 g/L KCl. A single-component additive (SH110 or SPS) was then added to the primary solution without a suppressor or leveller.

A silicon chip with TSV (diameter 20 μm and depth 60 μm) was used for electroplating. A blind via was etched by Bosch-type deep reaction ion etching (DRIE). A silicon dioxide layer of approximately 0.3 μm was deposited for sidewall insulation using tetraethoxysilane chemical vapor deposition (TEOS CVD), and then a copper seed layer was deposited through a physical vapor deposition (PVD) process.

During electrodeposition, the TSV sample was first immersed in deionized water in a vacuum chamber for 10 min, and then placed in an ultrasonicator for 60 s to remove air and impurities. After this pre-processing, the TSV sample was immersed in the plating solution for 10 min to allow the equilibration state to be reached between the additive and copper in the vias prior to electroplating. TSV filling was carried out with either SH110 or SPS additive in the primary solution, at a current density of 1 mA/cm^2^. After electrodeposition, the cross-section and microstructure of the TSVs were observed using SEM at 2000 × magnification (TESCAN MIRA3LMU).

### Electrochemical procedures

To study the electrochemical properties of the additives in the deposition process for TSV filling, LSV^19,20^ was performed on an electrochemical workstation (Chenhua CHI660E) using a three-electrode cell. The cell consisted of a 5-mm-diameter rotating Pt disk electrode (Pt-RDE) as the working electrode (WE), a Pt counter electrode (CE), and a saturated calomel electrode (SCE) as the reference. The testing potential ranged from 0.6 to 0.7 V versus Pt-RDE with a scan rate of 5 mV/s. The LSV measurements were performed using a potentiostat/galvanostat (PARSTAT 2273) at 25 °C.

### Theory and computational details

MD simulations were performed using Materials Studio software (https://www.3ds.com/products-services/biovia/ material studio 6.0)^21^ in order to understand the adsorption behaviour of SPS and SH110 on a Cu (111) surface in a simulation box (2.0448 × 1.0224 × 3.5435 nm) with periodic boundary conditions. These parameters allowed a representative part of the interface to be modelled without arbitrary boundary effects. The simulation box consisted of a copper slab with a 2.5-nm-high vacuum layer on top. The crystals in the slab were cut along the (111) plane, keeping the uppermost and lowest layers released and the inner layer fixed. The simulation was carried out using an NVT/NVE ensemble, where the system atomic number (N), volume (V), and energy (E) remained unchanged. The simulation was carried out below 298 K, with the time step and simulation time set to 0.1 fs and 500 ps, respectively. The force field COMPASS was used for the entire simulation procedure.

### Quantum chemistry calculations

Quantum chemical calculations^7,16,22^ were performed by the DMol^3^ module of Materials Studio. Complete geometries of the additives were fully optimised without any symmetry constraints using B3LYP/6-31G (d, p) with GAUSSIAN 09 W.

## Conclusions

The single-component additive SH110 was used for TSV copper electroplating, resulting in void-free TSV filling. The electrochemistry of SH110 indicates that it acts to both accelerate and suppress, with suppression that is better than that of the conventional additive, SPS. SH110 exhibits better bottom-up filling ability than SPS over a wider potential range. According to MD simulations, the main factor contributing to acceleration is the coplanar orientation of the MPS moiety of SH110 adsorbed to the copper surface. SH110 can be adsorbed to the Cu surface by the DHT and MPS moieties, while SPS—which essentially contains two MPS molecules—is adsorbed to the Cu surface only by MPS moieties. Quantum chemistry calculations show that in SH110, the DHT moiety provides an inhibitory effect for TSV filling, while the MPS acts as an accelerator. SH110 is therefore an excellent additive exhibiting both acceleration and suppression, which proves useful in achieving void-free TSV filling. Thus, we may use SH110 for TSV filling in 3D integration in further work.

## Data Availability

All data generated or analysed during this study are included in this published article.
